# Small RNA and degradome deep sequencing reveal respective roles of cold-related microRNAs across Chinese wild grapevine and cultivated grapevine

**DOI:** 10.1186/s12864-019-6111-5

**Published:** 2019-10-15

**Authors:** Pengfei Wang, Yang Yang, Hongmei Shi, Yongmei Wang, Fengshan Ren

**Affiliations:** 1 Shandong Academy of Grape; Shandong engineering research center for Grape cultivation and deep-processing, Jinan, People’s Republic of China; 2 Shandong engineering research center for cultivation and deep-processing of grape, Jinan, People’s Republic of China; 3 Key Laboratory of Urban Agriculture (East China), Ministry of Agriculture, Jinan, People’s Republic of China

**Keywords:** *Vitis amurensis*, *Vitis vinifera* L., miRNA, Cold tolerance, Orthologs

## Abstract

**Background:**

Chinese wild grapevine (*Vitis amurensis*) has remarkable cold stress tolerance, exceeding that of the common cultivated grapevine (*Vitis vinifera L*.).

**Result:**

Here, we surveyed the expression dynamics of microRNAs (miRNAs) across Chinese wild grapevine (cv. Beibinghong) and cultivated grapevine (cv. Cabernet Sauvignon) under cold stress using high-throughput sequencing. We identified 186 known miRNAs in cultivated grape and 427 known miRNAs in Beibinghong. Of the identified miRNAs, 59 are conserved miRNAs orthologous in Cabernet Sauvignon and Beibinghong. In addition, 105 and 129 novel miRNAs were identified in Cabernet Sauvignon and Beibinghong, respectively. The expression of some miRNAs was related to cold stress both in Cabernet Sauvignon and Beibinghong. Many cold-related miRNAs in Cabernet Sauvignon and Beibinghong were predicted to target stress response-related genes such as MYB, WRKY, bHLH transcription factor genes, and heat shock protein genes. However, the expression tendency under cold treatment of many of these miRNAs was different between Cabernet Sauvignon and Beibinghong. Different modes of expression of cultivated and Chinese wild grape miRNAs were indicated in key pathways under cold stress by degradome, target prediction, GO, and KEGG analyses.

**Conclusion:**

Our study indicated three likely reasons that led to the different cold stress tolerance levels of Cabernet Sauvignon and Beibinghong. Specifically, there may be (1) differential expression of orthologous miRNAs between cultivated grapevine and Chinese wild grape; (2) species-specific miRNAs or target genes; or (3) different regulatory models of miRNAs in cultivated and Chinese wild grape in some key pathways.

## Background

MicroRNA (miRNA) is an 18–24 nucleotide-long non-coding small RNA (sRNA) that can regulate gene expression by blocking protein translation through binding with the 3′ untranslated region (3’UTR) of the target gene mRNA in animals [1]. In plants, miRNAs can negatively regulate target gene mRNA by two major mechanisms: transcript cleavage and translational inhibition [2, 3]. MicroRNAs have important regulatory functions in the growth and development of plants. Moreover, miRNAs have been confirmed to be involved in various abiotic stress responses in plants, such as cold stress (chilling or freezing) [4–7].

Cold stress is known to impact the survival, productivity, and geographical distribution of plants [8]. Cold stress tolerance is a complex trait in plants [9, 10]. Cold stress could activate the Inducer of CBF Expression (ICE) gene, which is a type of basic helix-loop-helix (bHLH) gene, via the ABA-independent pathway [1, 11–14], and ICE may activate the downstream transcription factor C-repeat (CRT)-binding factors (CBF) or dehydration-responsive element binding (DREB) that binds to C-repeat elements (CRT)/low-temperature-response elements (LTRE) [1, 11]. Inducing the expression of downstream genes, including *cold-responsive* (*COR*) [10, 15], *CBF*, and *DREB*, could play critical roles in the cold acclimation process [10, 16]. MicroRNAs are also involved in the regulatory network of these proteins [1]. *COR* and other cold-regulated genes could produce two types of products. The first include heat shock proteins (HSP), antifreeze proteins, late embryogenesis abundant proteins (LEA), lipid transfer proteins, dehydrins, and compatible solutes such as raffinose, free sterols, sugars, glucosides, proline, and glycine betaine [11, 17–19]. The second products are various transcript factors (TFs). These TFs are involved in the regulation of signal transduction and the expression of downstream genes [20]. In addition, the abscisic acid (ABA) level in several cold-tolerant plant varieties was found to be significantly higher than in cold-sensitive varieties during cold acclimation [1].

The same study showed that ABA hormone signaling, which is involved in the cold stress response of herbaceous tissues, is controlled by various miRNAs [1]. The expression of many miRNAs was dynamic under cold stress in plants. For example, miR397 and miR169 were found to be up-regulated in Arabidopsis, *Brachypodium*, and poplar under cold stress [21]. MiR168 was up-regulated both in poplar and Arabidopsis [22], but miR168 was down-regulated in rice [23] during cold stress. Previous studies have shown that some miRNAs, such as miR394a, miR319, miR408, and miR396, were involved in the regulation of cold stress response in some plants [24–26]. For example, one previous study showed that 35S:At-miR394a OE Arabidopsis lines showed higher free proline levels and more total soluble sugars than wild type plants [24]. In addition to enhanced expression of other cold responsive genes (such as CBF1, CBF2, RD29A, and COR15a), an increased expression (up to 90-fold) of CBF3 was also observed in At-miR394a Arabidopsis OE lines [24]. Enhanced accumulation of free proline and increased expression of cold stress-related genes were detected in At-miR319 Arabidopsis OE lines [25]. The 35S:At-miR408 lines showed higher survival, higher optimal/maximal quantum yield of PSII (Fv/Fm) indicating the efficiency of photosystem II, low electrolyte leakage, and lower levels of MDA than At-miR408-knockout lines and wild type plants [26]. However, the orthologs of these miRNAs in grapevine were not shown to be involved in the regulation of cold stress response.

Cold stress is an important environmental factor that can negatively affect grapevine productivity and quality. Chinese wild grapevine (*Vitis amurensis* Rupr.) is more cold-tolerant than the common cultivated grapevine (*Vitis vinifera* L.) [27]. However, the molecular mechanisms that contribute to the enhanced cold stress tolerance of Chinese wild grapevine remain unknown. This study aimed to investigate the relationship between cold stress tolerance of Chinese wild grapevine and miRNA regulation. Here, sRNA and degradome deep sequencing were performed to detect and analyze cold-related miRNAs and their target genes in Chinese wild grapevine. Cabernet Sauvignon, a cold-sensitive grapevine cultivar (cv), was also examined to detect and analyze the cold-related miRNAs and their target genes.

## Results

### Data mining of small RNA sequencing

To reveal grape miRNAs related to cold, 12 small RNA libraries were constructed and sequenced. These samples included three cold-treated Cabernet Sauvignon sample repetitions (cold cultivated grape), three untreated Cabernet Sauvignon sample repetitions (CK cultivated grape), three cold-treated Beibinghong sample repetitions (cold Chinese wild grape), and three untreated Beibinghong sample repetitions (CK Chinese wild grape). After sequencing, a total of 364,979,306 raw and 334,324,103 clean reads were obtained. There was an average of 30,414,942 raw and 27,860,342 (91.61%) clean reads in each library. The clean reads were used to analyze the small RNAs. sRNAs of 20 nt long were the most abundant sRNA in both Cabernet Sauvignon and Beibinghong (Additional file 22: Figure S1). On average, 81.61 and 81.18% of clean reads from untreated Cabernet Sauvignon and cold-treated Cabernet Sauvignon samples, respectively, could be mapped to the grape genome database v2.0 (http://genomes.cribi.unipd.it/grape/). The average mapping of clean reads from untreated Beibinghong samples and cold-treated Beibinghong on the grape genome database were of 81.66 and 80.05%, respectively. Using the BLASTn and blastall search options against the GenBank and Rfam databases [28], all small RNAs identified from the 12 small RNA libraries were annotated, including rRNA, snoRNA, tRNA, and snRNA-derived (Table 1).

**Table 1:** Proportion of other small RNAs

Small RNA class	Proportion in small RNA class	
	untreated cultivated grape	cold-treated cultivated grape
rRNA-derived sRNA	1.07%	0.95%
snoRNA-derived sRNA	0.04%	0.03%
tRNA-derived sRNA	0.02%	0.01%
snRNA-derived sRNA	0.01%	0.01%
	untreated Chinese wild grape	cold-treated Chinese wild grapevine
rRNA-derived sRNA	0.82%	1.77%
snoRNA-derived sRNA	0.03%	0.06%
tRNA-derived sRNA	0.01%	0.03%
snRNA-derived sRNA	0.01%	0.01%

### Identification and expression patterns of known miRNAs

In Cabernet Sauvignon, we identified 186 known miRNAs, which were also deposited in grape miRNA data from the miRBase database (v20) (Additional file 1: Table S1). In Beibinghong, we identified 427 known plant miRNAs (Additional file 2: Table S2), of which 59 known miRNAs are conserved miRNAs orthologous in Cabernet Sauvignon and Beibinghong (only exhibiting one to three nucleotide differences in the sequences; Additional file 3: Table S3). For example, orthologous pairs include Chinese wild grape miR172a with cultivated grape Vvi-172a; Chinese wild grape miR159a with cultivated grape Vvi-159a; and Chinese wild grape miR160b with cultivated grape Vvi-miR160b.

In Cabernet Sauvignon, three known miRNAs were expressed only in the untreated library, and eight known miRNAs were expressed only in the cold-treated library. In cold-treated Cabernet Sauvignon, 41 known miRNAs were significantly (log2 fold change > 1 and *P* < 0.05) up-regulated and 33 known miRNAs were significantly (log2 fold change<− 1 and *P* < 0.05) down-regulated. In Cabernet Sauvignon, Vvi-miR395h was the most up-regulated by cold treatment, followed by Vvi-miR171c and Vvi-miR164a. Vvi-miR171a was the most down-regulated by cold treatment, followed by Vvi-miR395c and Vvi-miR395b. In Cabernet Sauvignon, most miR169, miR171, miR172, miR167, miR393, miR319, and miR396 family members were significantly (log2 fold change > 1 and *P* < 0.05) up-regulated by cold treatment. In Cabernet Sauvignon, most miR397 family members and miR408 were significantly (log2 fold change <− 1 and P < 0.05) down-regulated by cold treatment (Additional file 4: Table S4). In Beibinghong, 78 known miRNAs were expressed only in the untreated library and 77 known miRNAs were expressed only in the cold-treated library. In cold-treated Beibinghong, 116 known miRNAs were significantly (log2 fold change > 1 and *P* < 0.05) up-regulated and 109 known miRNAs were significantly (log2 fold change <− 1 and P < 0.05) down-regulated. In Beibinghong, miR3932b-5p was the most up-regulated by cold treatment, followed by miR156b-3p and miR3633a-3p; miR2118f was the most down-regulated by cold treatment, followed by miR171 and miR8036-3p. In Beibinghong, the miR169 family member miR169z was significantly up-regulated by cold treatment. In Beibinghong, miR171 and the miR172 family member miR172a were significantly down-regulated by cold treatment (Additional file 5: Table S5). In the 59 known orthologous miRNAs, 40 miRNAs had a different expression tendency between the cultivated grape and Chinese wild grape in the cold treatment (Additional file 6: Table S6).

### Identification of potentially novel miRNAs and analysis of their expression changes under cold stress

**Fig. 1:**
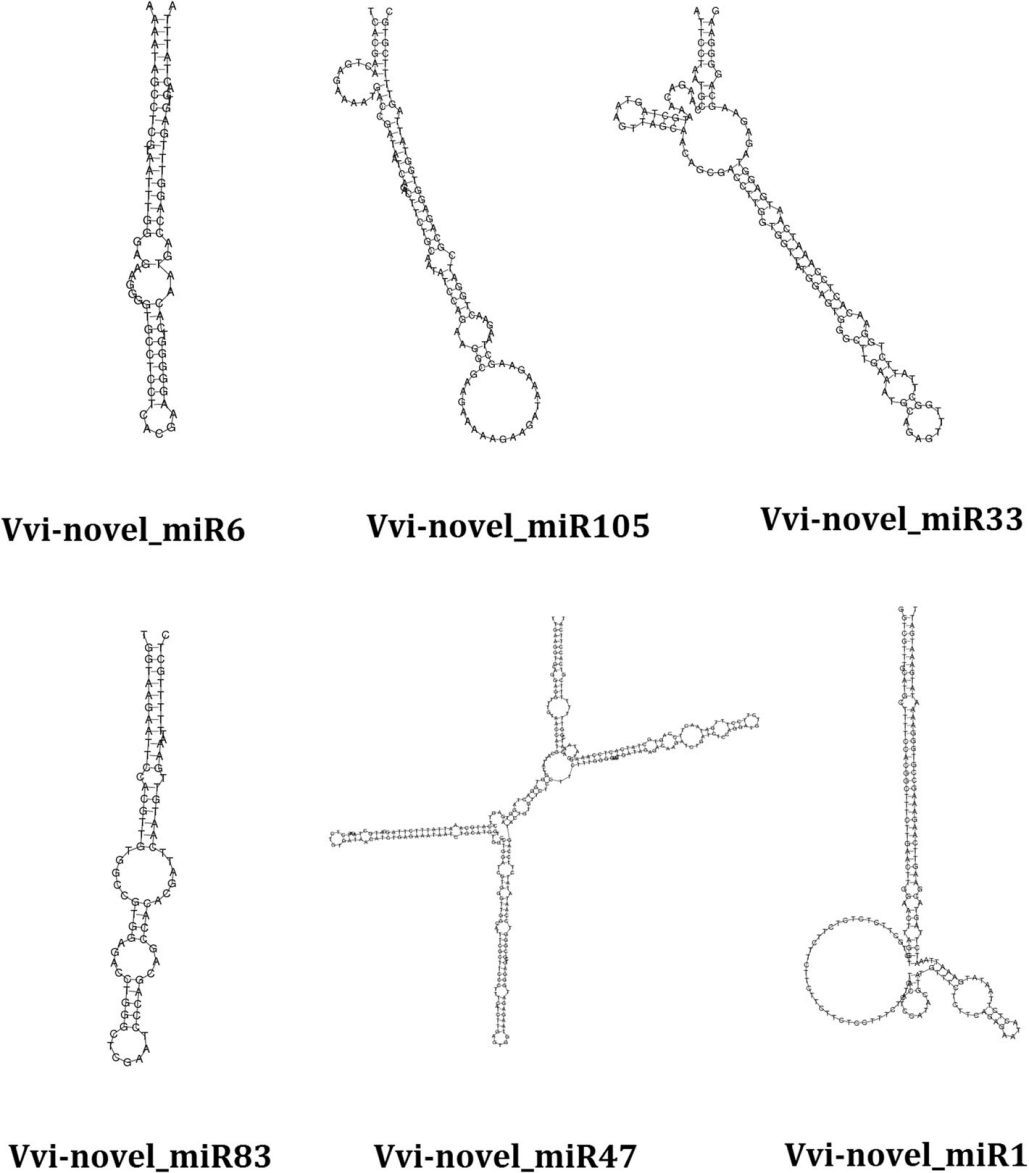
Examples of novel grape miRNA precursor structures

We identified 105 novel miRNAs and 129 novel miRNAs in cultivated grape and Chinese wild grape, respectively (Additional file 7: Table S7 and Additional file 8: Table S8). All of the miRNA complement sequences of these novel miRNAs were found in our study, and their mature sequences and star sequences are listed in Additional file 7: Table S7 and Additional file 8: Table S8. Some structures of novel miRNA precursors of cultivated grape or Chinese wild grape are listed in Fig. 1.

In Cabernet Sauvignon, no novel miRNAs were expressed only in the untreated library, and three novel miRNAs were expressed only in the cold-treated library. Thirty novel miRNAs were significantly (log2 fold change > 1 and *P* < 0.05) up-regulated and one novel miRNA was significantly (log2 fold change <− 1 and P < 0.05) down-regulated in cold-treated cultivated grape. In Cabernet Sauvignon, the novel miRNA Vvi-novel_mir69 was the most up-regulated by cold treatment, followed by Vvi-novel_mir47 and Vvi-novel_mir72. Vvi-novel_mir6 was the most down-regulated by cold treatment (Additional file 3: Table S3). No novel miRNAs were expressed in only the untreated and cold-treated Chinese wild grape. However, six novel miRNAs were significantly (log2 fold change > 1 and P < 0.05) up-regulated and two novel miRNAs were significantly (log2 fold change <− 1 and P < 0.05) down-regulated in cold-treated Beibinghong. In Beibinghong, novel_mir120 was the most up-regulated by cold treatment, followed by novel_mir73 and novel_mir25. Novel_mir55 was the most down-regulated in the cold treatment, followed by novel_mir79 and novel_mir98 in cold-treated Beibinghong (Additional file 4: Table S4).

### Target prediction and degradome analyses

In cultivated grape, a total of 1986 target genes (protein-coding genes) were predicted in silico (Additional file 9: Table S9). These genes could be targeted by 230 miRNAs. Our results showed that 85 differentially expressed cultivated grape miRNAs were predicted to target 925 protein-coding genes in silico. These differentially expressed cultivated grape miRNAs targeted AP2-like ethylene-responsive transcription factor genes, transcription factor HY5 genes, transcription factor TCP2 genes, squamosa promoter-binding-protein-like (SBP) genes, MYB transcription genes, transcription factor GAMYB genes, and heat shock protein 90.1 isoform 1 genes, which are stress-related genes. Differentially expressed cultivated grape miRNAs also targeted auxilin-like protein genes, auxin response factor 18 genes, and ETHYLENE INSENSITIVE 3-like genes, which are hormone signal transduction-related genes (Additional file 10: Table S10).

In Chinese wild grape, a total of 11,543 target genes (protein-coding genes) were predicted. In total, 499 Chinese wild grape miRNAs could target these genes (Additional file 11: Table S11). Our results showed that 212 differentially expressed Chinese wild grape miRNAs were predicted to target 4959 protein-coding genes (Additional file 12: Table S12), which targeted WRKY transcription genes, MYB transcription genes, ethylene-responsive transcription factor genes, bHLH transcription genes, transcription factor TCP2 genes, NAC transcription factor genes, SBP genes, GATA transcription factor genes, TIR-NB-LRR type resistance protein genes, heat shock cognate 70 kDa protein genes, and universal stress protein genes, which are stress-related genes. Differentially expressed miRNAs of Beibinghong also targeted auxin-induced protein genes and abscisic acid receptor genes as well as photosynthesis, amino acid, and sugar transporter related genes such as chlorophyll a-b binding protein genes, bidirectional sugar transporter genes, and cationic amino acid transporter genes (Additional file 12: Table S12).

The 40 differentially expressed miRNA orthologs were predicted to target 427 genes including NAC domain-containing protein genes, ethylene-responsive transcription factor genes, AP2-like ethylene-responsive transcription factor TOE3 genes, microtubule-associated protein genes, auxin transport protein genes, and promoter-binding protein genes (Additional file 13: Table S13).

**Fig. 2:**
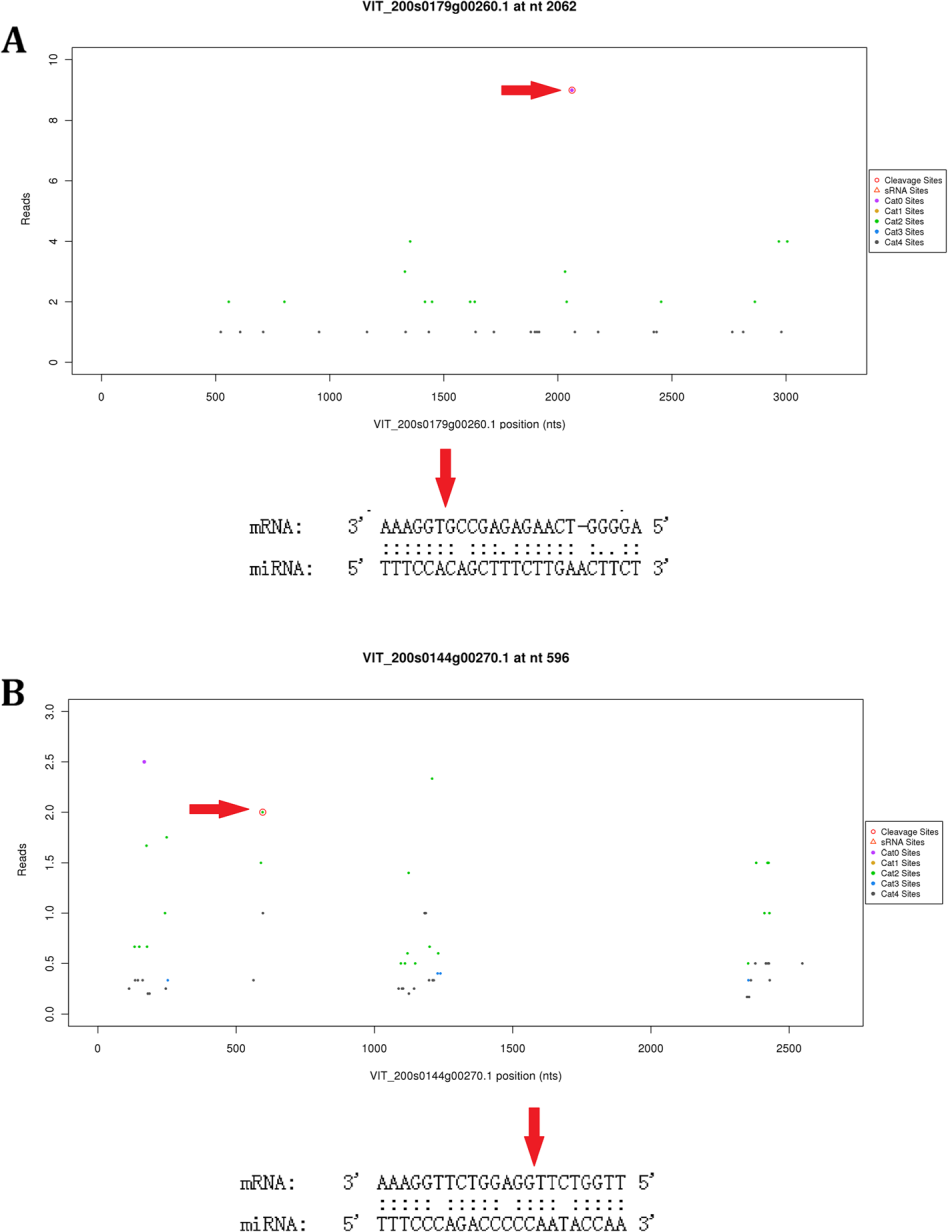
Target plots and miRNA–mRNA alignments. **a** Cultivated grape novel_miR9 cleaves VIT_200s0179g00260.1, (**b**) Vvi-miR3632-3p cleaves VIT_200s0144g00270.1

We employed degradome sequencing to identify miRNA targets. After sequencing, a total of 11,253,630, 12,596,309, 12,548,638, and 12,367,509 clean tags were obtained from untreated Cabernet Sauvignon, cold-treated Cabernet Sauvignon, untreated Beibinghong, and cold-treated Beibinghong, respectively. In cultivated grapevine, 141 target genes and 145 specific miRNA–mRNA pairs were identified using degradome sequencing (Additional file 14: Table S14). In Chinese wild grapevine, 359 target genes and 375 specific miRNA–mRNA pairs were identified using degradome sequencing (Additional file 15: Table S15). Target genes related to stimulus responses included WRKY transcription factor 47-like, heat shock cognate 70 kDa protein 2 gene cleaved by Chinese wild grape miR160b, and novel_mir64 (Additional file 15: Table S15). Fifty-one predicted target genes in silico were validated using degradome sequencing (36% of target genes identified using degradome sequencing) in cultivated grape. In silico, 124 predicted target genes were validated using degradome sequencing (35% of target genes identified using degradome sequencing) in Beibinghong. The target genes identified included miRNA–mRNA pair novel_mir9-*VIT_200s0179g00260.1* and vvi-miR3632-3p-*VIT_200s0144g00270.1* (Fig. 2). This showed that the in silico prediction of target genes was relatively reliable.

### GO enrichment and KEGG pathway analyses target differentially expressed miRNAs

**Fig. 3:**
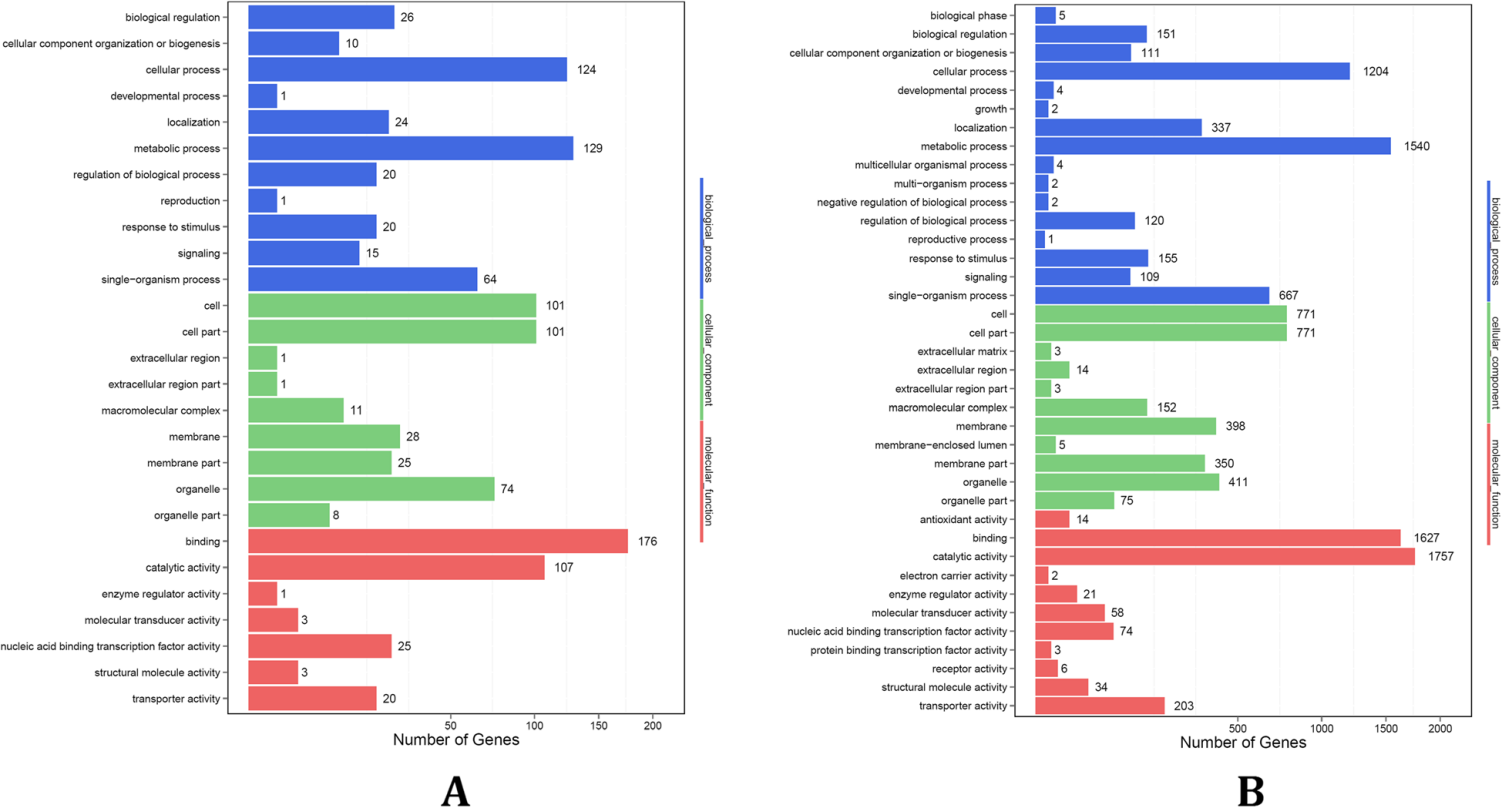
GO terms of targets of DEMs in (**a**) cultivated and (**b**) Chinese wild grape

Significantly (*P* < 0.05) up-regulated or down-regulated miRNAs were thought to be differential expressed miRNAs (DEMs) in our study. Gene ontology (GO) annotation and enrichment analysis was performed to further investigate the potential role of miRNAs in response to cold stress of two grapevine species. All targets predicted in silico and identified using degradome sequencing of differentially expressed cultivated grape miRNAs were classified into three categories: 714 in biological processes, 474 in molecular function, and 540 in cellular components. Biological processes contained 11 sub-categories including 171 terms, molecular functions contained seven sub-categories including 109 terms, and cellular components contained eight sub-categories including 44 terms (Fig. 3a, Additional file 16: Table S16). In biological processes, the significantly enriched (*P* < 0.05) GO terms were lignin metabolic process (GO: 0009808), secondary metabolic process (GO: 0019748), hormone-mediated signaling pathway (GO: 0009755), cellular response to hormone stimulus (GO: 0032870), heterocycle metabolic process (GO: 0046483), cellular nitrogen compound biosynthetic process (GO: 0034641), cellular amino acid biosynthetic process (GO: 0008652), and cellular aromatic compound metabolic process (GO: 0006725). In molecular functions, the significantly enriched (*P* < 0.05) GO terms were oxidoreductase activity (GO: 0016682), DNA binding (GO: 0003677), ATPase activity coupled to transmembrane movement of ions (GO: 0042625), P-P-bond-hydrolysis-driven transmembrane transporter activity (GO: 0015405), alcohol binding (GO: 0043178), amylase activity (GO: 0016160), and protein kinase activity (GO: 0004672). In cellular components, the significantly enriched (*P* < 0.05) GO terms were extracellular region (GO: 0005576), peroxisomal membrane (GO: 0005778), intrinsic to peroxisomal membrane (GO: 0031231), microbody membrane (GO: 0031903), intracellular membrane-bound organelle (GO: 0043231), and membrane-bound organelle (GO: 0043227; Additional file 17: Table S17).

In Chinese wild grape, the GO annotation and enrichment analysis identified 4417 target genes in biological processes, 2953 miRNAs in molecular function, and 3977 in cellular components. Biological processes contained 16 sub-categories including 510 terms, molecular functions contained 11 sub-categories including 331 terms, and cellular components contained 11 sub-categories including 108 terms (Fig. 3b, Additional file 18: Table S18). In biological processes, the significantly enriched (*P* < 0.05) GO terms were heterocycle metabolic process (GO: 0046483), trehalose metabolic process (GO: 0005991), primary metabolic process (GO: 0044238), cellular nitrogen compound metabolic process (GO: 0034641), cytoskeleton organization (GO: 0007010), cellular macromolecule metabolic process (GO: 0044260), cellular aromatic compound metabolic process (GO: 0006725), ribonucleoside triphosphate metabolic process (GO: 0009199), and anion transport (GO: 0006820). In molecular function, the significantly enriched (*P* < 0.05) GO terms were small molecule binding (GO: 0036094), P-P-bond-hydrolysis-driven transmembrane transporter activity (GO: 0015405), active transmembrane transporter activity (GO: 0022804), alcohol binding (GO: 0043178), sugar transmembrane transporter activity (GO: 0051119), and protein kinase activity (GO: 0004672). In cellular components, the significantly enriched (P < 0.05) GO terms were spindle (GO: 0005819), cytoskeletal part (GO: 0044430), intrinsic to membrane (GO: 0031224), and membrane part (GO: 0044425; Additional file 17: Table S17).

**Fig. 4:**
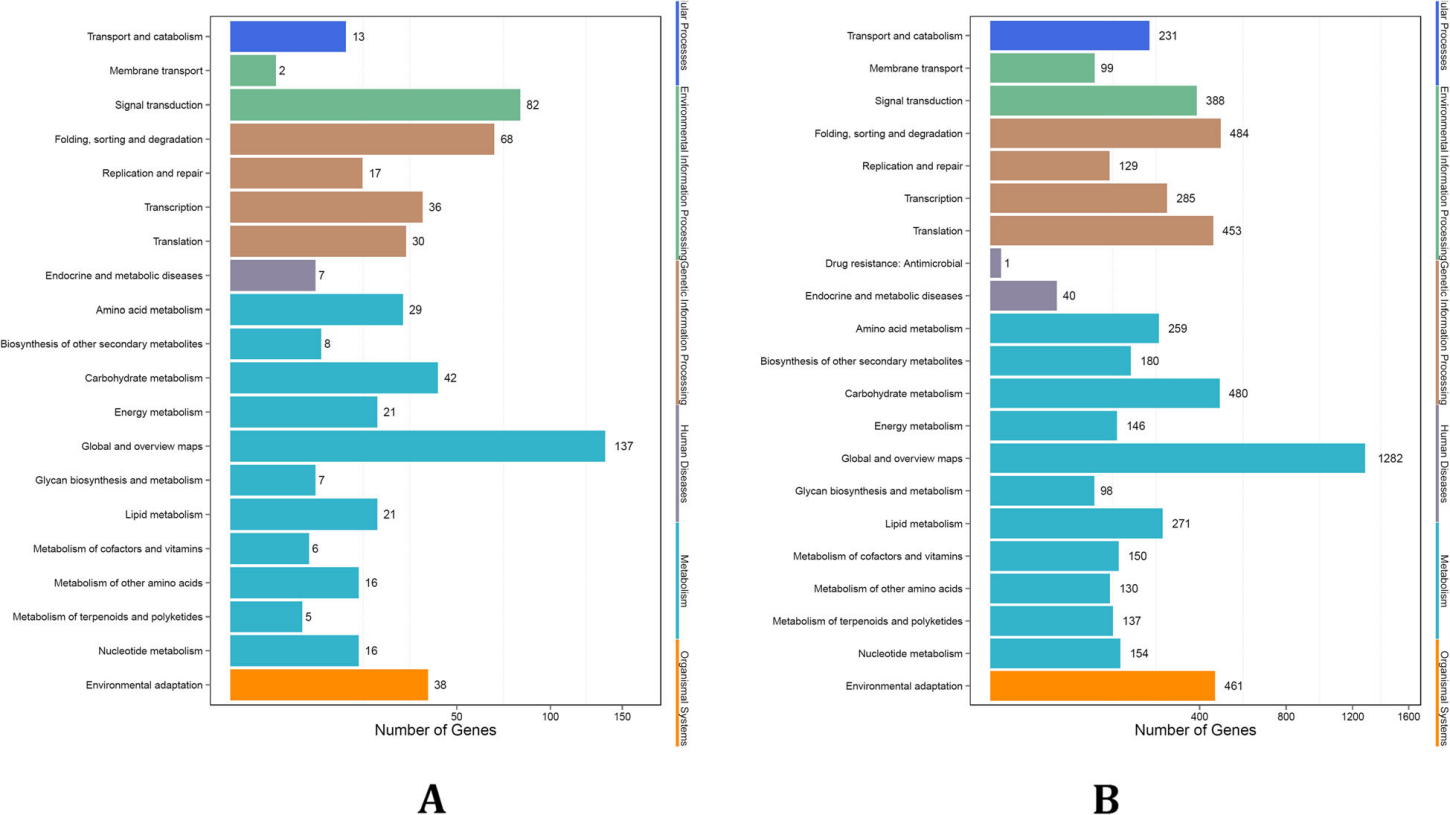
KEGG pathways of targets of DEMs in (**a**) cultivated and (**b**) Chinese wild grape

The KEGG annotation, enrichment analysis, and pathway annotation found that in target genes of differentially expressed miRNAs from cultivated grape, 38 were involved in environmental adaptation (Fig. 4a). In addition, 87 pathways were obtained, and the pathways that were found to be significantly enriched (P < 0.05) were plant hormone signal transduction (ko04075), DNA replication (ko03030), RNA degradation (ko03018), protein processing in endoplasmic reticulum (ko04141), and spliceosome (ko03040); Additional file 19: Table S19). In target genes of differentially expressed miRNAs from Chinese wild grape, 461 were involved in environmental adaptation (Fig. 4b). Of these, 134 pathways were obtained and the pathways that were significantly enriched (*P* < 0.05) were plant-pathogen interaction (ko04626), spliceosome (ko03040), ribosome biogenesis in eukaryotes (ko03008), RNA degradation (ko03018), and protein processing in endoplasmic reticulum (ko04141; Additional file 20: Table S20).

**Fig. 5:**
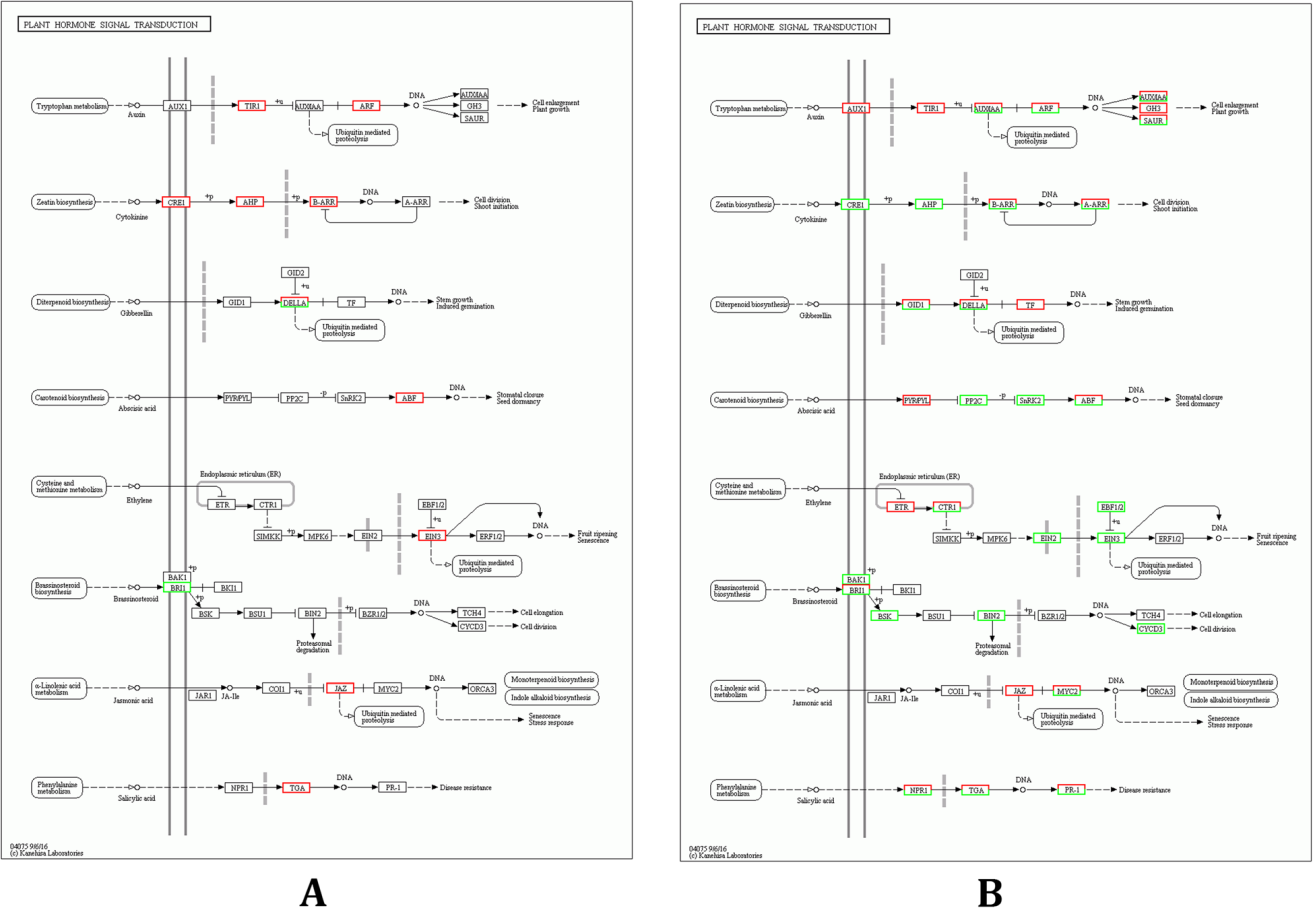
Plant hormone signal transduction pathway and related targets of DEMs in grape. **a** Plant hormone signal transduction pathway and related targets of DEM in cultivated grape. **b** Plant hormone signal transduction pathway and related targets of DEM in Chinese wild grape. The red box represents target gene-related miRNAs that were up-regulated and the green box represents target gene-related miRNAs that were down-regulated

**Fig. 6:**
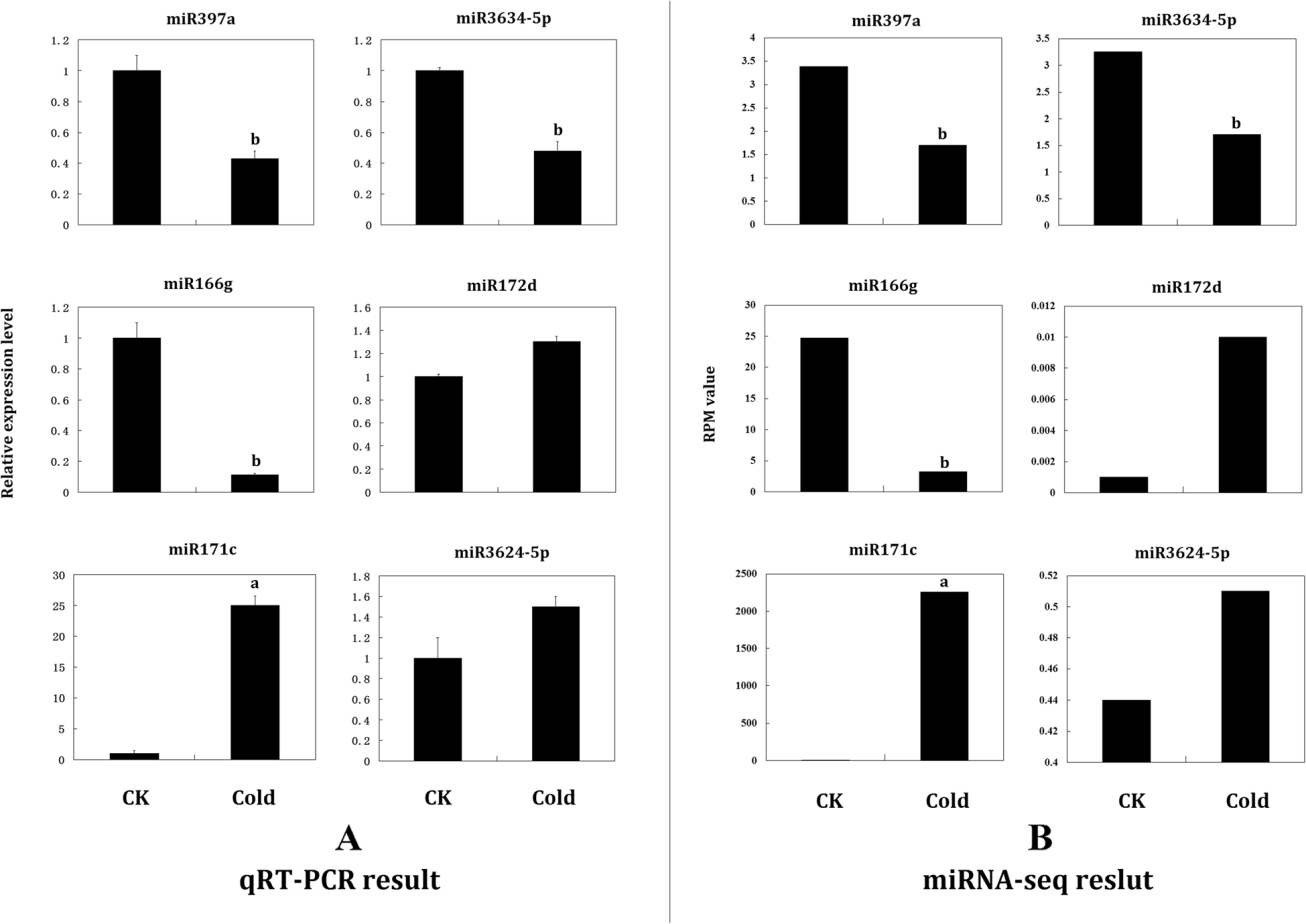
qRT-PCR validation of randomly selected miRNAs in cultivated grape. **a** The relative expression levels of the six miRNAs under cold treatment. **b** The RPM value of the six miRNAs under normal condition and cold treatment obtained from deep sequencing. “a” represents significantly up-regulated (*P* < 0.05) and “b” represents significantly down-regulated (*P* < 0.05)

In hormone signal transduction (Fig. 5) and starch and sucrose metabolism (Additional file 23: Figure S2), miRNAs found in Chinese wild grapevine appear to play different roles from those found in cultivated grape miRNAs in response to cold stress. In ABA hormone signal transduction pathways (Fig. 5), the cultivated grape miRNA Vvi-miR166a, which targeted ABF gene *VIT_208s0007g03420.1*, was up-regulated under cold stress. In Beibinghong, miR166a was not found. In Beibinghong, miR5234, miR7776-5p, miR166h-3p, and miR472b targeted ABF genes; however, these four miRNAs were down-regulated. In Chinese wild grape ABA hormone signal transduction pathways, miR6020a-5p, which targets the upstream gene of ABF gene, SNF1-related protein kinase genes (*SnRK2*; *VIT_218s0001g06310.2* and *VIT_218s0001g06310.1*) was down-regulated by cold treatment. Vvi-miR6020a-5p was not found in Cabernet Sauvignon. In Chinese wild grape JA hormone signal transduction pathways (Fig. 6), miR7755-3p and miR9408-3p, which targeted MYC genes *VIT_202s0025g03450.1–3* and *VIT_215s0046g00320.1*, were down-regulated. However, in Cabernet Sauvignon, the expression of miRNAs, which targeted MYC genes was unchanged. Chinese wild grape miRNAs novel_mir61 and miR396f, which targeted the EIN3 gene *VIT_200s0357g00120.1* and EIN2 genes *VIT_208s0040g01730.2*, respectively, were down-regulated in the ethylene signal transduction pathway. These two miRNAs were not found in Cabernet Sauvignon. The cultivated grape Vvi-miR3629a-3p, which targeted the D-glucose synthesis-related lysosomal beta glucosidase gene (*VIT_213s0073g00550.1*), was up-regulated under cold stress. In Beibinghong, miR3629a-3 was not up-regulated and could not target lysosomal beta glucosidase gene. miR5079a, miR6253, and miR9654a-3p, which, respectively, targeted the lysosomal beta glucosidase genes *VIT_207s0141g00250.1*, *VIT_216s0098g01760.4*, and *VIT_218s0089g00940.1*, were down-regulated in Beibinghong.

### qRT-PCR validation of miRNA expression

We performed qRT-PCR to validate the results of small RNA deep sequencing using six miRNAs from cultivated grape, Vvi-miR171c, Vvi-miR172d, Vvi-miR3624-5p, Vvi-miR166g, Vvi-miR397a, and Vvi-miR3634-5p. The primers used for the qRT-PCR are listed in Additional file 21: Table S21. qRT-PCR was performed using the Cabernet Sauvignon sample under control and cold conditions with three technical and three biological replicates. The expression results were similar to the deep sequencing data. Vvi-miR171c was significantly up-regulated in the qRT-PCR analysis, showing a positive correlation with the deep sequencing results. Vvi-miR166g was significantly down-regulated in both qRT-PCR and small RNA deep sequencing results. Other miRNAs were not significantly changed in either the qRT-PCR or small RNA deep sequencing results (Fig. 6).

## Discussion

As post-transcriptional gene regulators, miRNAs have been shown to be involved in the regulation of cold stress response in plants [24–26, 29]. For example, over-expression of miR397a could increase the transcript levels of cold-induced CBF (CBF1, CBF2, and CBF3) and downstream cold responsive genes in Arabidopsis [30]. In *Brachypodium*, miR397, miR172, miR171, miR169, and miR408 could be induced by cold stress [31]. In Arabidopsis, miR393 and miR397 could be induced by cold stress [4]. In poplar, miR167, miR171, and miR1450 could be induced by cold stress [32]. In sugarcane, miR319 could be induced by cold stress [33]. In cultivated grapevine cv. Muscat Hamburg, most miR171 family members, miR172 family members, miR172d, and most miR395 family members could be down-regulated by cold stress [34]. Investigations into cold-related miRNAs in cultivated grape and Chinese wild grape are limited; therefore, the aims of our research were to detect the cold-related miRNA in cultivated grape and Chinese wild grape to understand the roles of miRNAs in grape cold tolerance, and to compare their different functions in cold stress tolerance process between cultivated grape and Chinese wild grape.

### Differential expression of orthologous miRNAs between cultivated grapevine and Chinese wild grape may Lead to different cold stress tolerance

Here, we employed deep sequencing technology to systematically study the effect of cold stress on miRNA expression in cultivated grape and Chinese wild grape. We found that 59 known miRNAs are conserved miRNAs orthologous in cultivated grape and Chinese wild grape. Forty of the identified miRNA orthologs showed different expression tendencies between the two taxa when exposed to the cold treatment. The cultivated grapevine Vvi-miR171a was significantly down-regulated in the cold treatment (Additional file 4: Table S4). This is consistent with the changes in the expression of the miRNA in cultivated grapevine cv. Muscat Hamburg [34]. In Chinese wild grape, miRNAs orthologous miR171a were up-regulated by cold treatment. The target genes of Vvi-miR171a were F-box/LRR-repeat protein, scarecrow-like protein 22 isoform X2, squamosa promoter-binding-like protein 13A, and 7-deoxyloganetic acid glucosyltransferase gene. In cultivated grapevine, Vvi-miR172a was up-regulated under cold stress, and Vvi-miR164b and Vvi-miR535a were down-regulated under cold stress; the expression tendency of the miRNA orthologs in Chinese wild grape was the opposite. Vvi-miR535a, vvi-miR172a, and Vvi-miR164b targeted the late embryogenesis abundant (LEA) protein, AP2-like ethylene-responsive transcription factor, and NAC transcription factor gene, respectively in cultivated grapevine. The targets of these miRNAs in Chinese wild grape were the same as in cultivated grapevine. Some AP2-like ethylene-responsive transcription factors could interact with CRT/DER, increasing the expression level of some COR genes and the cold tolerance of plants [35]. Some LEA proteins have been shown to be involved in the freezing tolerance of plants [36]. Previous research has shown that some NAC transcription factors negatively regulated cold tolerance via the CBF-dependent pathway [37]. These orthologous miRNAs were cold-related and were differentially expressed. It is possible that differential expression of orthologous miRNAs between cultivated grapevine and Chinese wild grape may lead to the difference in their tolerance to cold stress.

### Species-specific miRNAs or target genes may Lead to different cold stress tolerance of cultivated grapevine and Chinese wild grape

The target of Chinese wild grape miR858a was MYB transcription factor, MYB3. Many MYB transcription factor genes have been shown to work together in the regulation of cold tolerance in plants [38–40]. The target of Chinese wild grape miR2109-5p was heat shock cognate 70 kDa protein gene. Heat shock proteins (HSPs) have also been shown to accumulate in response to low temperatures [41, 42]. The target of Chinese wild grape miR169a-5p was WRKY transcription gene *WRKY2*. WRKY transcription factors have been shown to be involved in modulating gene expression in plants during cold stress [43, 44]. The target of Chinese wild grape miR8179 was bHLH49 gene. Basic helix-loop-helix (bHLH) transcription factors positively modulate the CBF-pathway and confer tolerance to cold-stress in plants [45]. In Chinese wild grape, these miRNAs and target genes were identified to be cold-related. The above mentioned Chinese wild grape miRNAs were not found in cultivated grape.

Some orthologous miRNAs between cultivated grapevine and Chinese wild grape have species-specific target genes. For example, Chinese wild grape miR171a could target endonuclease III homolog 1, chloroplastic-like isoform X1, polycomb group protein EMBRYONIC FLOWER 2-like isoform X3, and glucose-1-phosphate adenylyltransferase large subunit 1 gene. However, cultivated grapevine Vvi-miR171a could not target these genes. These data are consistent with a previous study on cultivated grapevine cv. Muscat Hamburg [34]. We suspect that species-specific miRNAs or target genes led to the different cold stress tolerance levels of cultivated grapevine and Chinese wild grape.

### Different regulation models of miRNAs in key pathways may be essential to different cold stress tolerance

Results from the KEGG annotation showed that 38 targets of differentially expressed miRNAs of cultivated grape were involved in adaptation to the environment, and 460 targets of differentially expressed miRNAs of Chinese wild grape were involved in adaptation to the environment (Fig. 4). In view of the differences between the two cultivars of grapes indicated by the results of the KEGG annotation analysis, we analyzed the KEGG pathway location of targets of differentially expressed cultivated grape and Chinese wild grape miRNAs.

In the same pathways under cold stress, the miRNAs of the cultivated grape and Chinese wild grape may also play different roles. The ABA signal transduction pathway has been shown to be related to cold tolerance in plants [46]. In ABA signal transduction pathways (Fig. 5), the cultivated grape miRNA Vvi-miR166a, which targeted the ABA-responsive promoter element binding factor (ABF) gene *VIT_208s0007g03420.1*, was up-regulated under cold stress. In Chinese wild grape, miR166a was not found. Additionally, in Chinese wild grape, miR5234, miR7776-5p, miR166h-3p, and miR472b targeted ABF genes. However, these four miRNAs were down-regulated in cultivated grape. In Chinese wild grape ABA hormone signal transduction pathways, miR6020a-5p, which targeted the SNF1-related protein kinase gene (*SnRK2*; upstream of *ABF* and could increase the expression of *AB*F) was down-regulated by cold treatment. MiR6020a-5p was not found in cultivated grape. The up-regulation of Vvi-miR166a may degrade the ABF gene *VIT_208s0007g03420.1*, affecting the ABA signal transduction pathway and ABA-dependent cold response network under cold stress. In Chinese wild grape, the down-regulation of miR6020a-5p may be conducive to the accumulation of *SnRK2*, allowing the normal function of the ABA signal transduction pathway and regulating downstream genes related to cold tolerance [46]. JA positively regulates the C-REPEAT BINDING FACTOR (CBF) transcriptional pathway to up-regulate downstream cold-responsive genes and ultimately improve cold tolerance [47]. In Chinese wild grape JA hormone signal transduction pathways (Fig. 6), miR7755-3p and miR9408-3p, which targeted MYC genes *VIT_202s0025g03450.1–3* and *VIT_215s0046g00320.1*, were down-regulated. However, in cultivated grape, the expression of miRNAs targeted to *MYCs* was unchanged. MYC has been shown to be related to cold tolerance in plants and to play a key role in ABA-dependent cold response network [46]. This showed the miRNAs may be regulated to promote the accumulation of MYC genes and the JA signal transduction in Chinese wild grape but not in cultivated grape under cold stress. In the ethylene signal transduction pathway, the over-expression of *EIN3* has been shown to decrease cold tolerance in plants [48]. Interestingly, Chinese wild grape miRNAs novel_mir61 and miR396f, which targeted the EIN3 gene *VIT_200s0357g00120.1* and the EIN2 gene *VIT_208s0040g01730.2*, were down-regulated in the ethylene signal transduction pathway. These two miRNAs were not found in cultivated grape.

Our research showed that in the starch and sucrose metabolism pathway (Additional file 23: Figure S2), the cultivated grape miRNA Vvi-miR3629a-3p that targeted the D-glucose synthesis-related lysosomal beta glucosidase gene (*VIT_213s0073g00550.1*) was up-regulated under cold stress. In Chinese wild grape, miR3629a-3 was not up-regulated and could not target lysosomal beta glucosidase gene. In contrast, miR5079a, miR6253, and miR9654a-3p, which targeted the lysosomal beta glucosidase genes *VIT_207s0141g00250.1*, *VIT_216s0098g01760.4*, and *VIT_218s0089g00940.1*, respectively, were down-regulated in Chinese wild grape. Glucose can improve the cold tolerance of plants [11, 17–19]. This showed that miRNAs may be regulated to promote the accumulation of the D-glucose synthesis-related lysosomal beta glucosidase gene in Chinese wild grape, but not in cultivated grape under cold stress.

## Conclusions

In this study, we analyzed cold-related miRNAs and their target genes as well as the relationship between cold stress tolerance and miRNA regulation in two grapevine cultivars. We present three like explanations for the different cold stress tolerance levels of cultivated grapevine and Chinese wild grape, namely, (1) differential expression of orthologous miRNAs between cultivated grapevine and Chinese wild grape; (2) species-specific miRNAs or target genes; (3) different regulatory models of miRNAs in cultivated grapevine and Chinese wild grape in some key pathways.

## Methods

### Plant materials

The parents plants of Chinese wild grapevine cv. Beibinghong and cultivated grapevine cv. Cabernet Sauvignon used in this study were grown in the National Field Gene Bank for Amur Grapevine, Zuojia Town, Jilin Province, China and provided by Mr. Yiming Yang and Changyu Li. Chinese wild grapevine cv. Beibinghong was formally identified by Mr. Rungang Song and Wenpeng Lu (National Field Gene Bank for Amur Grapevine, Zuojia, China) and grapevine cv. Cabernet Sauvignon was formally identified by Baron Hector Brane (Chateau Mouton Rothschild, Pauillac, France). One-year-old self-rooted seedlings of grapevine cv. Cabernet Sauvignon and Chinese wild grapevine cv. Beibinghong were grown and maintained in the greenhouse under a 16-h light/8-h dark photoperiod at 26 °C. For the cold stress treatment, plant materials under a 16-h light/8-h dark photoperiod were transferred to 4 °C for 4 hours. For the control (CK), plants were kept under a 16-h light/8-h dark photoperiod at 26 °C for 4 hours. The shoot apices with leaves were collected from each treatment. Three biological replicates were used for the CK and cold treatment samples. RNA of each biological replicate was isolated, used for the construction of small RNA and degradome libraries, and were also use for real-time RT-PCR analysis.

### Construction of small RNA libraries and deep sequencing

RNAs were sent to BGI (Shenzhen, China) for small RNA library construction and high-throughput sequencing using BGISEQ-500 [49]. RNA of every biological replicate was isolated and was used in the construction of small RNA libraries for high-throughput sequencing.

### Identification of known and novel miRNAs using deep sequencing

The raw reads were collapsed and used as input for the web-based tool miRMaster [50], allowing for integrated analysis of miRNA data. Bowtie [51] was used to map the clean reads to the grape genome v2.0 [52] and miRNA data from the miRBase database (v20) [53] incorporating data from grape and other species (one mismatch allowed). The miRDeep2 algorithm was used to quantify miRNAs [54]. The prediction of novel miRNAs was performed using an extended set of eight features built using novoMiRank [55]. For classification, an AdaBoost model using decision trees was applied. Novel miRNAs were cross-checked against other RNA resources, including the miRBase [56], NONCODE2016 [57], and Ensembl non-coding RNAs. The assessment of the quality of new miRNAs was carried out using the novoMiRank algorithm [49]. The miRNAs with zero reads in all three biological replicates were considered absent. Otherwise, the miRNAs were considered present.

### Analysis of differentially expressed miRNAs

DEGseq [58] was used to identify the differentially expressed miRNAs based on an MA-plot [59]. Log2 fold change indicates the Log2 ratio (miRNA expression in cold/CK).

### Target prediction, GO, and KEGG pathway analyses

For target prediction, we applied psRobot [60] and TargetFinder [61] to predicted target genes of all miRNAs. Target genes were annotated based on the Gene Ontology database (http://www.geneontology.org/). Pathway analyses of target genes were performed using the KEGG (The Kyoto Encyclopedia of Gene and Genome) database (http:// www.genome.jp/kegg/kegg1.html) [62]. GO enrichment analysis was conducted according a previous study [28].

### Construction of the Degradome library, sequencing, and data analysis

The degradome library was constructed as previously described [63]. The degradome cDNA library was sequenced using an Illumina GAIIx. After sequencing, the adapter sequences, low-quality reads, and N-containing fragments were filtered from the raw reads. Then the clean tags could be reserved for the next analysis [28]. Clean tags were mapped to the grape genome allowing only two mismatches, and the analysis of cleavage sites and the classification of target categories were performed using CleaveLand 2.0 [28, 64].

### Validation of miRNA expressions with qRT-PCR analysis

The stem-loop quantitative RT-PCR (qRT-PCR) was performed to analyze the expression of miRNAs as previously described [65]. The U6 gene was used as the internal control. Primers used in all qRT-PCR experiments are listed in Additional file 21: Table S21.

## Supplementary information

**Additional file 1: Table S1.** Cultivated grape miRNA sequences.

**Additional file 2: Table S2.** Chinese wild grape miRNA sequences.

**Additional file 3: Table S3.** Conseved miRNA orthologous between cultivated grape and Chinese wild grape.

**Additional file 4: Table S4.** Differential expressed miRNAs in cultivated grape.

**Additional file 5: Table S5.** Differential expressed miRNAs in Chinese wild grape.

**Additional file 6: Table S6.** Expression changes of 40 differential expressed tendency’s miRNA orthologous in cultivated grape and Chinese wild grape.

**Additional file 7: Table S7.** Mature sequences, star sequences and precursor sequences of cultivated grape novel miRNAs.

**Additional file 8: Table S8.** Mature sequences, star sequences and precursor sequences of Chinese wild grape novel miRNAs.

**Additional file 9: Table S9.** Target genes of cultivated grape miRNAs.

**Additional file 10: Table S10.** Target genes of cultivated grape DEMs.

**Additional file 11: Table S11.** Target genes of Chinese wild grape miRNAs.

**Additional file 12: Table S12.** Target genes of Chinese wild grape DEMs.

**Additional file 13: Table S13.** Target genes of 40 differential expressed tendency’s miRNA orthologous.

**Additional file 14: Table S14.** Target genes of cultivated grape miRNAs identified based on degradome sequencing.

**Additional file 15: Table S15.** Target genes of Chinese wild grape miRNAs identified based on degradome sequencing.

**Additional file 16: Table S16.** GO terms of targets of DEMs in cultivated grape.

**Additional file 17: Table S17.** Significantly enriched GO terms of targets of DEMs in cultivated and Chinese wild grape.

**Additional file 18: Table S18.** GO terms of targets of DEMs in Chinese wild grape.

**Additional file 19: Table S19.** KEGG pathways of targets of DEMs in cultivated grape.

**Additional file 20: Table S20.** KEGG pathways of targets of DEMs in Chinese wild grape.

**Additional file 21: Table S21.** Primers of randomly selected miRNAs for reverse transcription and qRT-PCR.

**Additional file 22: Figure S1.** Read counts of different lengths of small RNAs in cultivated grape (CK and cold treatment samples) and Chinese wild grape (CK and cold treatment samples).

**Additional file 23: Figure S2.** Starch and sucrose metabolic pathways and related targets of DEMs in grape**.** (a) Starch and sucrose metabolic pathways and related targets of DEM in cultivated grape. (b) Starch and sucrose metabolic pathways and related targets of DEM in Chinese wild grape. The red box represents target gene-related miRNAs that were up-regulated and the green box represents target genes that were down-regulated.

## Data Availability

The data sets supporting the results of this article are included within the article and its additional files. The sequencing data was available at SRA database with accession number of PRJNA563136 (https://dataview.ncbi.nlm.nih.gov/object/PRJNA563136?reviewer=460f3r82rd2gkkl0qmfu0qs50g).

## Abbreviations

3′UTR: 3′ untranslated region
ABA: abscisic acid
CBF: C-repeat-binding factors
COR gene: cold-responsive gene
DEM: differential expressed miRNA
DREB: dehydration-responsive element binding
HSP: heat shock proteins
ICE gene: Inducer of CBF Expression gene
LEA: late embryogenesis abundant proteins
LTRE: low-temperature-response elements
miRNA: MicroRNA
SBP gene: squamosa promoter-binding-protein-like gene
sRNA: small RNA
TF: transcript factors
