# Supplementary material for: Small RNA and degradome deep sequencing reveal respective roles of cold-related microRNAs across Chinese wild grapevine and cultivated grapevine
Source: BMC Genomics. 2019 Oct 15;20:740. doi: 10.1186/s12864-019-6111-5 (PMC6794902; doi:10.1186/s12864-019-6111-5)
Supplement: Supplementary file 3 — Additional file 3: Table S3. Conseved miRNA orthologous between cultivated grape and Chinese wild grape. [file 12864_2019_6111_MOESM3_ESM.doc]

**Table S3 Conseved miRNA orthologous between cultivated grape** and Chinese wild grape

| **Chinese wild** |  | **Cultivated grape** |  |
| --- | --- | --- | --- |
| **grape miRNA id** | **miRNA seq** | **miRNA id** | **miRNA seq** |
| miR159a | TTTGGATTGAAGGGAGCTCT | vvi-miR159a | CTTGGAGTGAAGGGAGCTCTC |
| miR160b | TGCCTGGCTCCCTGTATGCC | vvi-miR160b | TGCCTGGCTCCCTGAATGCCATC |
| miR162 | TTGATAAACCTCTGCATCCA | vvi-miR162 | TCGATAAACCTCTGCATCCAG |
| miR164b | TGGAGAAGCAGGGCACGTGC | vvi-miR164b | TGGAGAAGCAGGGCACATGCT |
| miR167d | TGAAGCTGCCAGCATGATCT | vvi-miR167d | TGAAGCTGCCAGCATGATCTA |
| miR168 | TCGCTTGGTGCAGGTCGGGA | vvi-miR168 | TCGCTTGGTGCAGGTCGGGAA |
| miR171a | TGATTGAGCCGTGCCAATAT | vvi-miR171a | TGATTGAGCCGTGCCAATATC |
| miR172a | AGAATATTGGATGATGCTGA | vvi-miR172a | TGAATCTTGATGATGCTACAT |
| miR2111-5p | TAATCTGCATCCTGAGGTCT | vvi-miR2111-5p | TAATCTGCATCCTGAGGTCTA |
| miR2950-3p | TGGTGTGCACGGGATGGAAT | vvi-miR2950-3p | TGGTGTGCACGGGATGGAATA |
| miR2950-5p | TTCCATCTCTTGCACACTGG | vvi-miR2950-5p | TTCCATCTCTTGCACACTGGA |
| miR3623-3p | TGGTGCTTGGACGAATTTGC | vvi-miR3623-3p | TGGTGCTTGGACGAATTTGCTA |
| miR3623-5p | TCACAAGTTCATCCAAGCACC | vvi-miR3623-5p | TCACAAGTTCATCCAAGCACCA |
| miR3624-3p | TCAGGGCAGCAGCATACTAC | vvi-miR3624-3p | TCAGGGCAGCAGCATACTACT |
| miR3624-5p | TAGTATGCTGCTGTCTTTAG | vvi-miR3624-5p | TAGTATGCTGCTGTCTTTAGA |
| miR3625-3p | CGGGAGATGACTACTGGAAG | vvi-miR3625-3p | CGGGAGATGACTACTGGAAGC |
| miR3625-5p | TTCCAGCAGTCATCTCCAAG | vvi-miR3625-5p | TTCCAGCAGTCATCTCCAAGG |
| miR3626-3p | TTCAATTTCACAGCGACCAC | vvi-miR3626-3p | CTTCAATTTCACAGCGACCAC |
| miR3626-5p | TAGTCGCTGTGAAATTGAAG | vvi-miR3626-5p | GGTAGTCGCTGTGAAATTGAA |
| miR3627-3p | TCGCCGCTCTCCTGTGACAA | vvi-miR3627-3p | TCGCCGCTCTCCTGTGACAAG |
| miR3627-5p | TCCCAGGAGAGATGGCACCTG | vvi-miR3627-5p | TTGTCGCAGGAGAGACGGCACT |
| miR3629a-3p | TGGCTGCTGAGAAAATGTAG | vvi-miR3629a-3p | GGCTGCTGAGAAAATGTAGGA |
| miR3629a-5p | CGCATTTTCTCAGCAGCCAA | vvi-miR3629a-5p | CGCATTTTCTCAGCAGCCAAG |
| miR3630-3p | TTTGGGAATCTCTCTGATGCA | vvi-miR3630-3p | TTTGGGAATCTCTCTGATGCAC |
| miR3630-5p | GCAAGTGACGATATCAGACAG | vvi-miR3630-5p | TGCAAGTGACGATATCAGACA |
| miR3631a-3p | TATTGGATGATGTCAACAGG | vvi-miR3631a-3p | TATATTGGATGATGTCAACAA |
| miR3631b-3p | TGTTGGTTGATGTCAATGAG | vvi-miR3631b-3p | TGTTGGATGATGTCAATAAGT |
| miR3632-3p | TTTCCCAGACCCCCAATACCA | vvi-miR3632-3p | TTTCCCAGACCCCCAATACCAA |
| miR3632-5p | GGATTGGGGGCCGATGGAAAG | vvi-miR3632-5p | GGATTGGGGGCCGATGGAAAGG |
| miR3633a-3p | TTCCTATACCACCCATTCCCT | vvi-miR3633a-3p | TTCCTATACCACCCATTCCCTA |
| miR3633a-5p | GGAATGGATGGTTAGGAGA | vvi-miR3633a-5p | GGAATGGATGGTTAGGAGAG |
| miR3633b-3p | GTTCCCATGCCATCCATTCCT | vvi-miR3633b-3p | GTTCCCATGCCATCCATTCCTA |
| miR3634-3p | TTTCCGACTCGCACTCATGCCG | vvi-miR3634-3p | TTTCCGACTCGCACTCATGCCGT |
| miR3634-5p | GGCATATGTGTGACGGAAAG | vvi-miR3634-5p | GGCATATGTGTGACGGAAAGA |
| miR3635-3p | ATTATGTCCCACACATGCCT | vvi-miR3635-3p | ATTATGTCCCACACATGCCTC |
| miR3635-5p | GGCATGTATGGGGCATAATA | vvi-miR3635-5p | GGCATGTGTGGGGCATAATAG |
| miR3636-3p | GTCTGTCGGAGAAGCAAGTCGGA | vvi-miR3636-3p | GTCTGTCGGAGAAGCAAGTCGGAG |
| miR3636-5p | TCGGTTTGCTTCTTTGATAGATT | vvi-miR3636-5p | TCGGTTTGCTTCTTTGATAGATTC |
| miR3637-3p | CGACAAGACACAATGCATAAATG | vvi-miR3637-3p | TTTCGACAAGACACAATGCATAAA |
| miR3637-5p | ATTTATGTATTGTGTTTTGTCGG | vvi-miR3637-5p | ATTTATGTATTGTGTTTTGTCGGA |
| miR3638-5p | TGTGCCTTTTCGCGCTTGTTGCT | vvi-miR3638-5p | TGTGCCTTTTCGCGCTTGTTGCTA |
| miR3639-3p | GAGCTTTTGGCTTCTCAGAAGTC | vvi-miR3639-3p | GAGCTTTTGGCTTCTCAGAAGTCA |
| miR3639-5p | TTGACTTCTGAAAGGCTAAAAGC | vvi-miR3639-5p | ATTGACTTCTGAAAGGCTAAAAGC |
| miR3640-3p | ATCGAAAAGGCATCATCAATCAG | vvi-miR3640-3p | ATCGAAAAGGCATCATCAATCAGG |
| miR3640-5p | TGATTGGTGATGCTTTTTTGGTA | vvi-miR3640-5p | ACCTGATTGGTGATGCTTTTTTGG |
| miR393a | TCCAAAGGGATCGCATTGATC | vvi-miR393a | TCCAAAGGGATCGCATTGATC |
| miR394a | TTGGCATTCTGTCCACCTC | vvi-miR394a | TTGGCATTCTGTCCACCTCCAT |
| miR395a | CTGAAGTGTTTGGGGGAACT | vvi-miR395a | CTGAAGTGTTTGGGGGAACTC |
| miR397a | TCATTGAGTGCAGCGTTGAT | vvi-miR397a | TCATTGAGTGCAGCGTTGATG |
| miR398b | TGTGTTCTCAGGTCGCCCCT | vvi-miR398b | TGTGTTCTCAGGTCGCCCCTG |
| miR399a | TGCCAAAGGAGAATTGCCCT | vvi-miR399a | TGCCAAAGGAGAATTGCCCTG |
| miR399e | CGCCAAAGGAGAGTTGCCCT | vvi-miR399e | TGCCAAAGGAGATTTGCCCGG |
| miR477a | ACTCTCCCTCAAGGGCTTCT | vvi-miR477a | ATCTCCCTCAAAGGCTTCCAA |
| miR477b-3p | CGAAGTCTTTGGGGAGAGTG | vvi-miR477b-3p | CGAAGTCTTTGGGGAGAGTGG |
| miR479 | TGTGGTATTGGTTCGGCTCAT | vvi-miR479 | TGTGGTATTGGTTCGGCTCATC |
| miR535a | TGACAACGAGAGAGAGCACG | vvi-miR535a | TGACAACGAGAGAGAGCACGC |
| miR828a | TCTTGCTCAAATGAGTATTCC | vvi-miR828a | TCTTGCTCAAATGAGTATTCCA |
| miR845a | CGCGCTCTGATACCACTTGT | vvi-miR845a | TAGCTCTGATACCAATTGATA |
| miR845d | TGCGCTCTGATACCACTTGT | vvi-miR845d | TGGCTCTGATACCAATTGATG |
