# Supplementary material for: Small RNA and degradome deep sequencing reveal respective roles of cold-related microRNAs across Chinese wild grapevine and cultivated grapevine
Source: BMC Genomics. 2019 Oct 15;20:740. doi: 10.1186/s12864-019-6111-5 (PMC6794902; doi:10.1186/s12864-019-6111-5)
Supplement: Supplementary file 17 — Additional file 17: Table S17. Significantly enriched GO terms of targets of DEMs in cultivated and Chinese wild grape. [file 12864_2019_6111_MOESM17_ESM.doc]

**Table S17 Significantly enriched GO terms of targets of DEMs in cultivated and Chinese wild grape**

Significantly enriched GO terms of targets of DEMs in cultivated grape

| **GO ID** | **GO term** | **P value** |
| --- | --- | --- |
| **Biological processes** | |  |
| GO:0009808 | lignin metabolic process | 1.30E-30 |
| GO:0009698 | phenylpropanoid metabolic process | 1.70E-24 |
| GO:0019748 | secondary metabolic process | 1.70E-24 |
| GO:0006725 | cellular aromatic compound metabolic process | 2.17E-17 |
| GO:1901360 | organic cyclic compound metabolic process | 3.49E-17 |
| GO:0034654 | nucleobase-containing compound biosynthetic process | 9.11E-14 |
| GO:0044271 | cellular nitrogen compound biosynthetic process | 1.08E-12 |
| GO:0018130 | heterocycle biosynthetic process | 2.18E-12 |
| GO:0019438 | aromatic compound biosynthetic process | 2.28E-12 |
| GO:1901362 | organic cyclic compound biosynthetic process | 2.49E-12 |
| GO:1901576 | organic substance biosynthetic process | 2.24E-10 |
| GO:0010033 | response to organic substance | 3.29E-10 |
| GO:0044249 | cellular biosynthetic process | 5.15E-10 |
| GO:0009725 | response to hormone stimulus | 6.67E-10 |
| GO:0009058 | biosynthetic process | 6.81E-10 |
| GO:0009719 | response to endogenous stimulus | 9.48E-10 |
| GO:0006351 | transcription, DNA-dependent | 1.01E-09 |
| GO:0032774 | RNA biosynthetic process | 1.01E-09 |
| GO:0071704 | organic substance metabolic process | 1.99E-07 |
| GO:0044237 | cellular metabolic process | 9.01E-07 |
| GO:0009755 | hormone-mediated signaling pathway | 1.46E-06 |
| GO:0032870 | cellular response to hormone stimulus | 1.46E-06 |
| GO:0070887 | cellular response to chemical stimulus | 1.46E-06 |
| GO:0071310 | cellular response to organic substance | 1.46E-06 |
| GO:0071495 | cellular response to endogenous stimulus | 1.46E-06 |
| GO:0090304 | nucleic acid metabolic process | 2.08E-06 |
| GO:0090407 | organophosphate biosynthetic process | 6.43E-06 |
| GO:0006139 | nucleobase-containing compound metabolic process | 8.25E-06 |
| GO:0016070 | RNA metabolic process | 8.99E-06 |
| GO:0009145 | purine nucleoside triphosphate biosynthetic process | 4.46E-05 |
| GO:0009206 | purine ribonucleoside triphosphate biosynthetic process | 4.46E-05 |
| GO:0009987 | cellular process | 4.69E-05 |
| GO:0009201 | ribonucleoside triphosphate biosynthetic process | 5.67E-05 |
| GO:0009142 | nucleoside triphosphate biosynthetic process | 6.13E-05 |
| GO:0042221 | response to chemical stimulus | 0.00012 |
| GO:0006807 | nitrogen compound metabolic process | 0.00012 |
| GO:0046483 | heterocycle metabolic process | 0.00016 |
| GO:0034641 | cellular nitrogen compound metabolic process | 0.00023 |
| GO:0070838 | divalent metal ion transport | 0.00043 |
| GO:0072511 | divalent inorganic cation transport | 0.00043 |
| GO:0008654 | phospholipid biosynthetic process | 0.00083 |
| GO:0034645 | cellular macromolecule biosynthetic process | 0.00125 |
| GO:0008152 | metabolic process | 0.00174 |
| GO:0009059 | macromolecule biosynthetic process | 0.00232 |
| GO:1901293 | nucleoside phosphate biosynthetic process | 0.00373 |
| GO:0044710 | single-organism metabolic process | 0.0318 |
| GO:0008652 | cellular amino acid biosynthetic process | 0.04184 |
| **Molecular function** | |  |
| GO:0016682 | oxidoreductase activity, acting on diphenols and related substances as donors, oxygen as acceptor | 9.33E-27 |
| GO:0016679 | oxidoreductase activity, acting on diphenols and related substances as donors | 3.65E-26 |
| GO:0001071 | nucleic acid binding transcription factor activity | 4.02E-13 |
| GO:0003899 | DNA-directed RNA polymerase activity | 1.98E-12 |
| GO:0005515 | protein binding | 2.26E-10 |
| GO:0003677 | DNA binding | 3.21E-06 |
| GO:0043178 | alcohol binding | 4.59E-06 |
| GO:0019829 | cation-transporting ATPase activity | 6.10E-06 |
| GO:0042625 | ATPase activity, coupled to transmembrane movement of ions | 1.77E-05 |
| GO:0016160 | amylase activity | 7.21E-05 |
| GO:0003676 | nucleic acid binding | 0.00012 |
| GO:0042626 | ATPase activity, coupled to transmembrane movement of substances | 0.00024 |
| GO:0043492 | ATPase activity, coupled to movement of substances | 0.00024 |
| GO:0016820 | hydrolase activity, acting on acid anhydrides, catalyzing transmembrane movement of substances | 0.00029 |
| GO:0005488 | binding | 0.00057 |
| GO:0004133 | glycogen debranching enzyme activity | 0.00078 |
| GO:0015399 | primary active transmembrane transporter activity | 0.00324 |
| GO:0015405 | P-P-bond-hydrolysis-driven transmembrane transporter activity | 0.00324 |
| **Cellular component** | |  |
| GO:0005576 | extracellular region | 1.84E-16 |
| GO:0005778 | peroxisomal membrane | 0.00044 |
| GO:0031231 | intrinsic to peroxisomal membrane | 0.00044 |
| GO:0031903 | microbody membrane | 0.00044 |
| GO:0043231 | intracellular membrane-bounded organelle | 0.00079 |
| GO:0043227 | membrane-bounded organelle | 0.00508 |

Table 4 Significantly enriched GO terms of targets of DEMs in Chinese wild grape

| **GO ID** | **GO term** | **P value** |
| --- | --- | --- |
| **Biological processes** | |  |
| GO:0034641 | cellular nitrogen compound metabolic process | 3.76E-05 |
| GO:0006139 | nucleobase-containing compound metabolic process | 9.76E-05 |
| GO:0046483 | heterocycle metabolic process | 0.0001 |
| GO:0006725 | cellular aromatic compound metabolic process | 0.0004 |
| GO:0009987 | cellular process | 0.00052 |
| GO:0044260 | cellular macromolecule metabolic process | 0.00056 |
| GO:0044237 | cellular metabolic process | 0.00064 |
| GO:0043412 | macromolecule modification | 0.00134 |
| GO:0006807 | nitrogen compound metabolic process | 0.00141 |
| GO:0007010 | cytoskeleton organization | 0.00197 |
| GO:0006464 | cellular protein modification process | 0.00387 |
| GO:0036211 | protein modification process | 0.00387 |
| GO:0044238 | primary metabolic process | 0.0082 |
| GO:1901360 | organic cyclic compound metabolic process | 0.01143 |
| GO:0005991 | trehalose metabolic process | 0.01565 |
| GO:0006753 | nucleoside phosphate metabolic process | 0.01793 |
| GO:0009199 | ribonucleoside triphosphate metabolic process | 0.02924 |
| GO:0009141 | nucleoside triphosphate metabolic process | 0.03175 |
| GO:0006793 | phosphorus metabolic process | 0.03863 |
| GO:0006796 | phosphate-containing compound metabolic process | 0.03902 |
| GO:0006820 | anion transport | 0.04548 |
| **Molecular function** | |  |
| GO:1901363 | heterocyclic compound binding | 1.78E-12 |
| GO:0097159 | organic cyclic compound binding | 1.87E-12 |
| GO:0016772 | transferase activity, transferring phosphorus-containing groups | 5.41E-09 |
| GO:0016301 | kinase activity | 2.21E-07 |
| GO:0036094 | small molecule binding | 4.19E-07 |
| GO:0000166 | nucleotide binding | 9.12E-07 |
| GO:1901265 | nucleoside phosphate binding | 9.12E-07 |
| GO:0032559 | adenyl ribonucleotide binding | 7.99E-06 |
| GO:0030554 | adenyl nucleotide binding | 8.39E-06 |
| GO:0034061 | DNA polymerase activity | 2.66E-05 |
| GO:0003899 | DNA-directed RNA polymerase activity | 3.78E-05 |
| GO:0004672 | protein kinase activity | 0.00012 |
| GO:0032553 | ribonucleotide binding | 0.00014 |
| GO:0032555 | purine ribonucleotide binding | 0.00014 |
| GO:0017076 | purine nucleotide binding | 0.00014 |
| GO:0016740 | transferase activity | 0.00035 |
| GO:0022804 | active transmembrane transporter activity | 0.00035 |
| GO:0015399 | primary active transmembrane transporter activity | 0.00077 |
| GO:0015405 | P-P-bond-hydrolysis-driven transmembrane transporter activity | 0.00077 |
| GO:0016773 | phosphotransferase activity, alcohol group as acceptor | 0.00186 |
| GO:0017111 | nucleoside-triphosphatase activity | 0.00247 |
| GO:0015923 | mannosidase activity | 0.00288 |
| GO:0043178 | alcohol binding | 0.01121 |
| GO:0005488 | binding | 0.01122 |
| GO:0015154 | disaccharide transmembrane transporter activity | 0.01177 |
| GO:0015157 | oligosaccharide transmembrane transporter activity | 0.01177 |
| GO:0005215 | transporter activity | 0.02634 |
| GO:0016818 | hydrolase activity, acting on acid anhydrides, in phosphorus-containing anhydrides | 0.0333 |
| GO:0022857 | transmembrane transporter activity | 0.0341 |
| GO:0042623 | ATPase activity, coupled | 0.03696 |
| GO:0016765 | transferase activity, transferring alkyl or aryl (other than methyl) groups | 0.04263 |
| GO:0016817 | hydrolase activity, acting on acid anhydrides | 0.04489 |
| GO:0008408 | 3'-5' exonuclease activity | 0.04642 |
| GO:0051119 | sugar transmembrane transporter activity | 0.04642 |
| GO:0016861 | intramolecular oxidoreductase activity, interconverting aldoses and ketoses | 0.04643 |
| **Cellular component** | |  |
| GO:0005819 | spindle | 1.48E-05 |
| GO:0044430 | cytoskeletal part | 1.48E-05 |
| GO:0031224 | intrinsic to membrane | 0.00079 |
| GO:0044425 | membrane part | 0.0304 |
