# Supplementary figures and images for: Small RNA and degradome deep sequencing reveal respective roles of cold-related microRNAs across Chinese wild grapevine and cultivated grapevine
Source: BMC Genomics. 2019 Oct 15;20:740. doi: 10.1186/s12864-019-6111-5 (PMC6794902; doi:10.1186/s12864-019-6111-5)

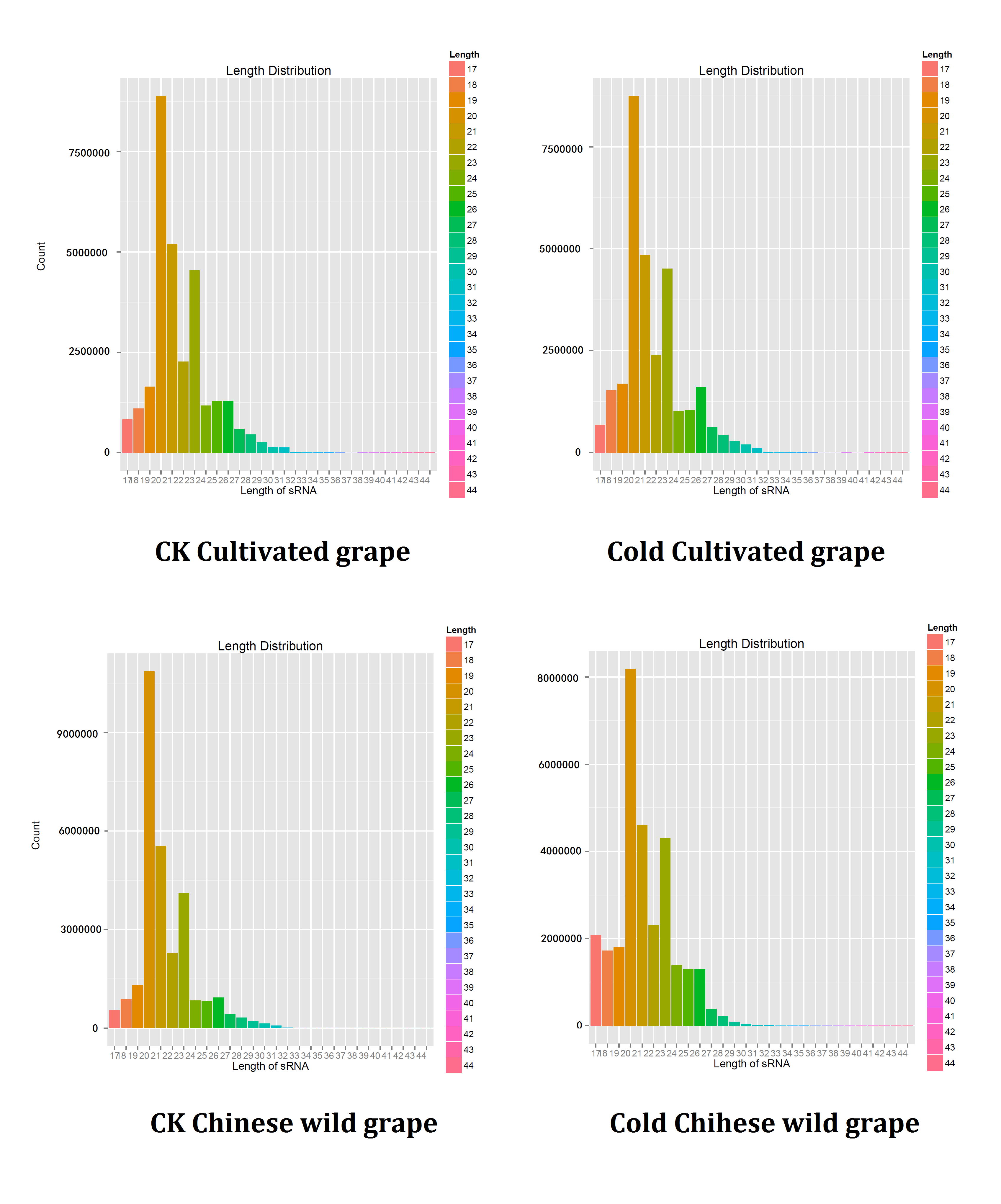

Supplement: Supplementary file 22 — Additional file 22: Figure S1. Read counts of different lengths of small RNAs in cultivated grape (CK and cold treatment samples) and Chinese wild grape (CK and cold treatment samples). [file 12864_2019_6111_MOESM22_ESM.tif]

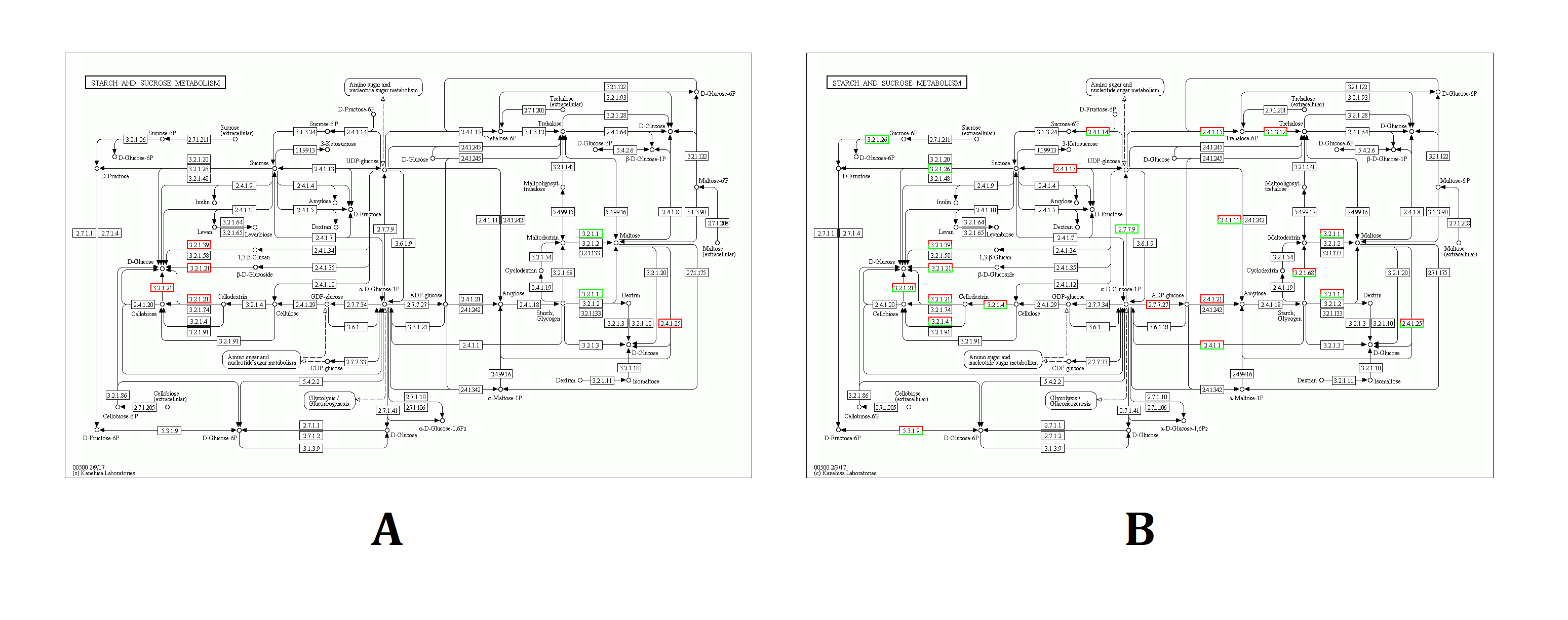

Supplement: Supplementary file 23 — Additional file 23: Figure S2. Starch and sucrose metabolic pathways and related targets of DEMs in grape. (a) Starch and sucrose metabolic pathways and related targets of DEM in cultivated grape. (b) Starch and sucrose metabolic pathways and related targets of DEM in Chinese wild grape. The red box represents target gene-related miRNAs that were up-regulated and the green box represents target genes that were down-regulated. [file 12864_2019_6111_MOESM23_ESM.tif]
